# Acute abdomen in adult revealing unusual complicated epiploic appendagitis: A case report

**DOI:** 10.1016/j.ijscr.2020.09.041

**Published:** 2020-09-11

**Authors:** Y. Charifi, Y. Lamrani, L. Chbani, M. Maaroufi, B. Alami

**Affiliations:** a Service de Radiologie, CHU HASSAN II Fès, faculté de medecine et de pharmacie, université Sidi Mohammed Ben Abdellah, Fez, Morocco; b Service d’anatomie et de cytologie pathologique, CHU HASSAN II Fès, Faculté de médecine et de pharmacie, Université Sidi Mohammed Ben Abellah, Fez, Morocco

**Keywords:** Epiploic appendagitis, Abscess, CT scan

## Abstract

**Introduction:**

Epiploic appendagitis is a torsion of fatty appendages of ligamentum trees (1), it’s a rare cause of abdominal pain that usually manifests by right or left iliac fossa pain, reminding of appendicitis, diverticulitis or ischemia of the omentum.

**Case presentation:**

We report the case of a 56 years old male patient admitted for an epigastric pain and a right-upper quadrant abdominal pain, for whom he underwent an abdominal ultrasound and a CT-scan who shown an abscessed mass under colic transverse to the unusual seat.

**Discussion:**

CT scan is still the key modality for diagnosis, avoiding unnecessary surgery. Complications of primary epiploic appendagitis have rarely been described in the literature (Hwang et al., 2013; Hasbahceci et al., 2012). Another more rare complication is appendagitis with associated abscess, in this condition, we must also think about complicated diverticulitis.

The treatment of uncomplicated forms is generally conservative, however, some authors have suggested a surgical method (Saad et al., 2014).

For therapeutic management of its complication, there has been much interest in the use of minimally invasive techniques such as percutaneous drainage to minimize the morbidity and mortality that is associated with surgery (Evidence National Guideline Centre (UK), 2019).

**Conclusion:**

The diagnosis of epiploic appendagitis is still uncommon, CT scan can eliminate other diagnosis of acute abdominal pain such as diverticulitis and appendicitis. It also allows the diagnosis of the rare complicated forms such as abscesses associated with epiploic appendagitis.

The treatment of typical forms is usually conservative, while the complicated form requires surgery because of the potential associated pathology.

## Highlights

• Think about the epiploic appendagitis more frequently when dealing with an obese patient with predispositions.
• Understand the importance of imagery that avoids unnecessary surgery in case of uncomplicated epiploic appendagitis and make it possible to do a radiological drainage.
• Always make the necessary differential diagnoses before evoking appendagitis.

## 1 Introduction

The epiploic appendix is a fatty structure covered with peritoneum and containing vessels from the colonic vascularization [1]. There are about 100 in all, more common are on the sigmoid and cecum. They are more prevalent and larger in obese patients [2].

Primary epiploic appendagitis is frequent [3] and corresponds to the inflammation of an epiploic appendix spontaneously or potentially by torsion or ischaemia.

The clinical and biological aspects are non-specific [4]. They often suggest ileocaecal appendicitis or diverticulitis.

Our work is focused on an uncommon complication of epiploic appendagitis which is abscess and it follows perfectly the SCARE criteria [11].

## 2 Case presentation

56 years old male, obese, smoker since 20 years, without occupation, admitted in our structure for epigastric and right hypochondrium pain progressing since one week with a 5 days of sub occlusive syndrome. The initial clinical examination showed epigastric and right hypochondrium sensibility associated with a fever reaching 38.5°.

Biology revealed hyperleukocytosis at 13,000 elements/mm3 associated with a raised C reactive protein at 57 mg/l.

**Fig. 1:**
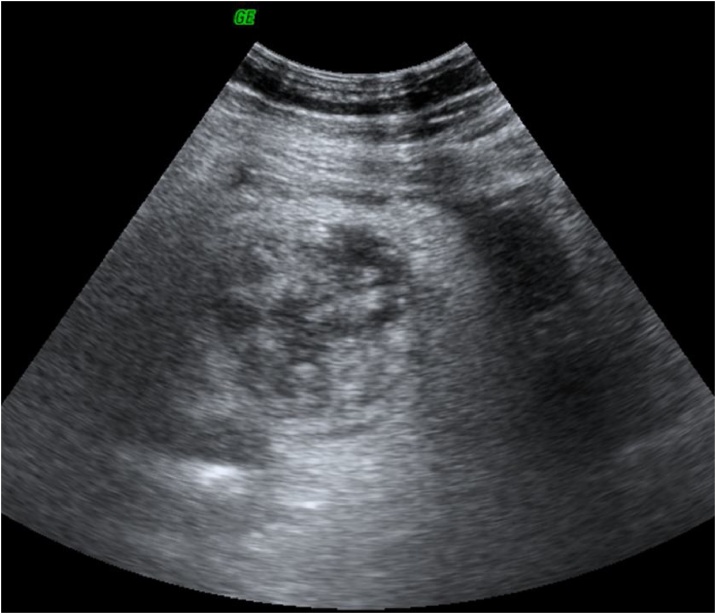
Ultrasound picture (using a low-frequency probe) showing a low echogenic, heterogeneous, thick-walled regular formation with a significant infiltration of the adjacent area.

An emergency abdominal ultrasound examination was performed and revealed a left supra-umbilical pseudo-mass, containing multiple partitions with significant fat and infiltration all around (Fig. 1).

**Fig. 2:**
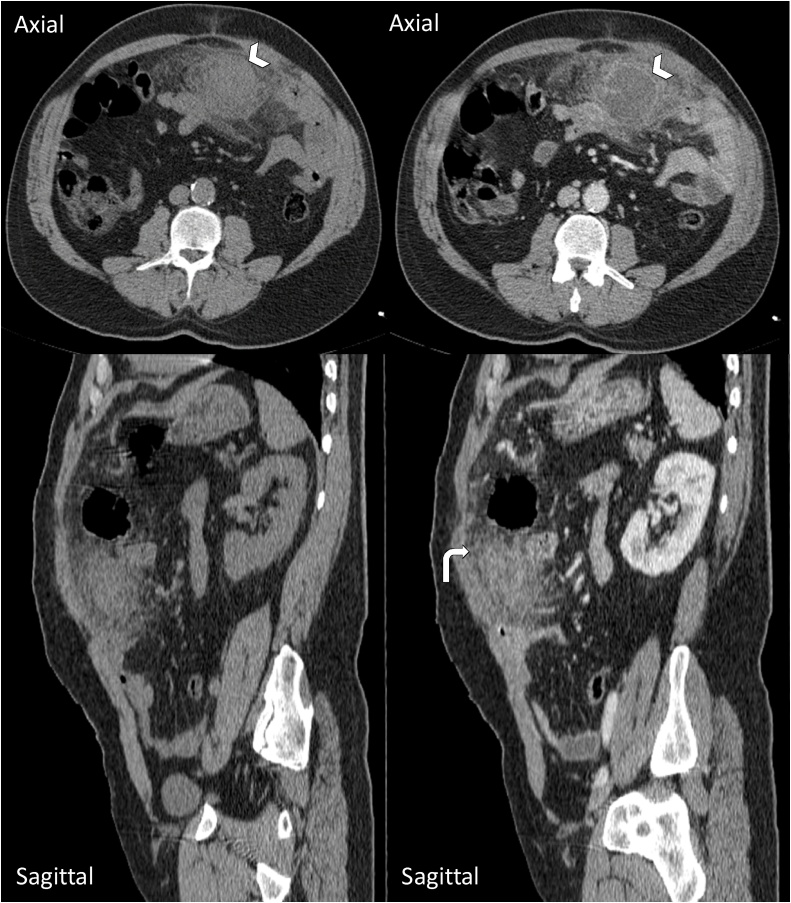
CT Scan images in axial and sagittal views before and after injection of contrast agent showing a supra-umbilical formation (arrowhead), rounded, with low density, and regular wall, enhanced after contrast, containing some fine septa. It is associated with significant local infiltration (curved arrowhead).

The patient had an abdominal CT scan with contrast injection, which showed a rounded formation at the expense of the large supra-umbilical omentum slightly lateralized on the left, under the transverse colon, which had a low-density, with thin septas inside, a regular wall and peripheral contrast enhancement. It is associated with a significant local infiltration. The appendix had a normal morphology: there was no intestinal occlusion (Fig. 2).

The surgeons indicated an exploration by midline laparotomy, and not radiological drainage, considering the clinical peritonitis and also the suspicion of ulcer perforation due to the patient's smoking history, even without pneumoperitoneum, its revealed an inflammatory epiploic mass, associated with a single microbial abscess, without any abnormalities of the gastrointestinal wall, so surgical excision of the mass was performed, and then the surgical piece was sent for anatomo-pathological study. The post-operative status was normal without immediate or late complications; the patient received antibiotic therapy with good clinical evolution.

One week after his hospitalization, the biological results improved with a hemoglobin level from 10.3 to 12 g/dl, leukocytes from 13,000 elements/mm3 to 11,000 elements/mm3 without significant variation in other parameters.

The microbiological results of the collection revealed that it was a sterile abscess, with no isolated germ.

**Fig. 3:**
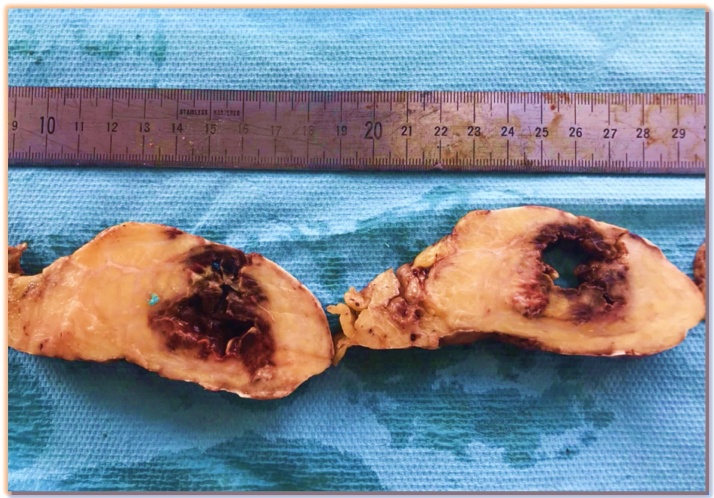
Macroscopy: fibrino-leukocyte layer.

**Fig. 4:**
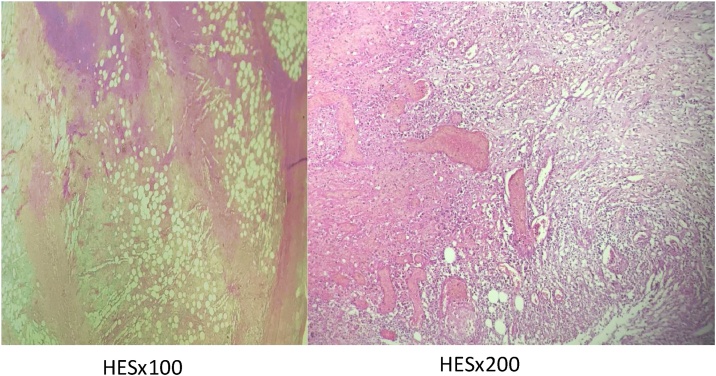
Microscopy: damaged fibrin and polynuclear tissue around fat.

The anatomopathological study of the pseudo-mass showed a macroscopic appearance of a fibrinoleukocyte layer (Fig. 3). The microscopic study showed altered fibrin and polynuclear tissue around the fat (Fig. 4).

## 3 Discussion

Epiploic appendagitis also known as “appendicitis epiploica” or “appendagitis,” results from an inflammation of the epiploïc appendix and is usually characterized by acute abdominal pain with localized defense. This symptomatology is due to the torsion-necrosis of an epiploïc fringe [5], which is a small (0.5–5 cm length, 1–2 cm wider) sub peritoneal fat formation composed of a duplication of the visceral peritoneum covering the colon. It involves the entire colon, including 50–100 appendixes which are distributed in order of frequency as follows: recto sigmoid (57%), ileocaecal area (26%), ascending colon (9%), transverse colon (6%), and descending colon (2%) [8].

The vascularization of an epiploic fringe is provided by 1 or 2 small arteries. The pedicular and mobile nature of these epiploic fringes leads to torsion on their axis, resulting to vessel thrombosis, ischemia and necrosis. Thrombosis of the own vessels can also occur and induce the same ischemic and necrotic effects [5,6,8].

In pathophysiology, a distinction is made between primary appendagitis when torsion or thrombosis occurs spontaneously and secondary appendagitis when it results from the spread of inflammation from nearby organs, especially in cases of diverticulitis, appendicitis or pancreatitis [7].

It is a rare pathology frequently diagnosed by laparotomy or laparoscopy [5]. The mean age of appearance is 35 years with a sex ratio close to 1. The clinical signs are not specific. The anamnesis reports constant pain, which may be of the grinding or colic type. Diarrhea is found in 25% of cases, moderate fever in 15% of cases [4]. Clinical examination reveals intense pain on palpation or even localized defense as we seen in our case [3]. In most cases, this clinical presentation may mimic other pathological conditions, such as acute appendicitis and diverticulitis [16].

Atypical signs such as respiratory symptoms can be found in cases of appendagitis on the transverse colon as observed in our study or at the angles of the colon, and exceptionally may simulate clinical forms of cholecystitis [4,5].

Biologically, appendagitis is not associated with biological changes such as hyperleukocytosis or inflammatory syndrome, however changes in these parameters may not necessarily mean that the diagnosis is excluded, but they may suggest complications such as an epiploic abscess, as in the current case [4,8,9].

Our patient was obese with a body mass index (BMI) of 30 kg/m2, so the ultrasound examination was difficult, however, we visualized a pseudo-mass, ovoid, non-compressible in the painful area, surrounded by a hyperechoic peripheral halo, and these aspects were compatible with those described in the literature [5].

CT scan is still the key modality for diagnosis, avoiding unnecessary surgery, by showing on unenhanced CT images a solitary mass adjacent to the colon, ovoid in a “shuttle” shape, with a higher density than normal peritoneal fat, surrounded by a high-density border [1 mm thickness] that shows inflammation of the visceral peritoneum covering the epiploic appendage, a peripheral fat infiltration is usually associated with the mass.

A central high density focus can be seen as a “dot sign” appearance, which is highly suggestive of venous thrombus within an inflamed epiploic appendage [7,8]. Post-contrast CT images do not show any enhancement of the epiploic appendagitis or adjacent peritoneum.

Generally, the treatment of uncomplicated forms is conservative, and requires an observation for 24 h, analgesic and hypocaloric diet. However, some authors have suggested that the surgical method of ligation and excision of inflamed epiploid appendages is the only way to prevent recurrence [16].

Complications of primary epiploic appendagitis have rarely been described in the literature [14,15]. In fact, the inflammatory process can sometimes cause adhesions and lead to obstruction of the small bowel and therefore require surgical treatment. Another more rare complication is appendagitis with associated abscess, in this condition the diagnosis of epiploic appendagitis secondary to complicated diverticulitis should be sought. For therapeutic management of this complication, there has been much interest in the use of minimally invasive techniques such as percutaneous drainage to minimize the morbidity and mortality that is associated with surgery. However, no clear guidance is currently available to suggest which patients should undergo percutaneous drainage versus surgery [12,13]. Although, laparoscopy or midline laparotomy are necessary on unexpected diagnosis if acute appendicitis or diverticulitis were suspected such as in our case [8,16,17].

An anatomo-pathological study of the operating epiploic appendagitis with abscess usually reveals inflammatory changes, the presence of fibrin and neutrophil polynuclear cells, with associated peritoneal reaction including congestion, oedema, and bleeding in the peripheral fat area; also some fibrinoleukocyte exudates on the surface of abcess [10].

## 4 Conclusion

The diagnosis of epiploic appendagitis is still uncommon, however, it is important that a clinician can consider it based on the different imaging modalities available especially abdominal CT scan, which allows to eliminate other conditions presenting as acute abdominal pain such as diverticulitis and appendicitis. It also allows the diagnosis of the rare complicated forms such as abscesses associated with epiploic appendagitis.

The treatment of typical forms is usually conservative, while the complicated form requires surgery because of the potential associated pathology.

## Declaration of Competing Interest

The authors report no declarations of interest.

## Source of funding

No source of funding was received.

## Ethical approval

Our study is exempt from ethical approval.

## Consent

Written informed consent was obtained from the patient for publication of this case report and accompanying images. A copy of the written consent is available for review by the Editor-in-Chief of this journal on request.

## Author contribution

CY, and HH performed and interpreted the patients CT scan and ultrasound. OA’s service did surgical procedure on the patient and the data are led to the pathology performed by CL, and the professor AB was a major contributor in writing the manuscript. All authors read and approved the final manuscript.

## Registration of research studies

NA.

## Guarantor

Dr Charifi Yahya.

Pr Alami Badreddine.

## Provenance and peer review

Not commissioned, externally peer-reviewed.
